# The effect on incisional hernia of absorbable barbed suture for midline fascial closure in minimally invasive surgery for colorectal and gastric cancers: study protocol for a randomized controlled trial

**DOI:** 10.1186/s13063-023-07324-x

**Published:** 2023-05-06

**Authors:** Sejin Lee, Se Wung Han, Min Ro Lee, Chan-Young Kim, Gi Won Ha

**Affiliations:** 1 Research Institute of Clinical Medicine of Jeonbuk National University-Biomedical Research Institute of Jeonbuk National University Hospital, Jeonju, Republic of Korea; 2 Department of Surgery, Jeonbuk National University Hospital, 20 Geonji-Ro, Deokjin-Gu, Jeonju, 54907 Republic of Korea

**Keywords:** Barbed suture, Fascia closure, Minimally invasive surgery, Randomized controlled trial

## Abstract

**Background:**

Incisional hernia following abdominal surgery is a frequent complication of midline laparotomy. This complication is strongly associated with the technique and material used for suture. While a monofilament absorbable suture is recommended to prevent incisional hernia, it can lead to suture loosening or surgical-knot breakage. Although barbed sutures can be an alternative suture material in abdominal fascial closure, evidence for its safety and effectiveness is lacking. Therefore, we designed a prospective randomized trial to evaluate the safety and efficacy of absorbable barbed sutures for midline fascia closure in minimally invasive surgery for colorectal and gastric cancers in comparison with conventional absorbable monofilament sutures.

**Methods:**

A total of 312 patients who underwent minimally invasive surgery for colorectal and gastric cancers will be randomly allocated to either the absorbable barbed or monofilament suture group for abdominal fascia closure in a 1:1 ratio. The primary outcome is incisional hernia rate within 3 years after surgery, as verified by physical examination and computed tomography. Postoperative complications, including surgical site infection, postoperative pain, and quality of life, will be compared between two groups as secondary outcomes. The investigator will examine the patients until discharge and at 6, 12, 18, 24, and 36 months postoperatively.

**Discussion:**

This is the first randomized controlled trial to compare absorbable barbed sutures with monofilament sutures for midline fascia closure in minimally invasive surgery. If absorbable barbed sutures demonstrate superior results to those of monofilament sutures, this type of suture material may be recommended as an alternative option for abdominal fascia closure.

**Trial registration:**

KCT0007069. Registered on January 30, 2023

## Introduction

### Background and rationale {6a, 6b}

Laparoscopic and robotic surgery has become a preferred treatment option for colorectal and gastric cancers rather than open surgery because of its minimally invasive nature and short-term operative outcomes [1–5]. During minimally invasive gastrointestinal surgery, mini-laparotomy is used for extracorporeal anastomosis, specimen extraction, or single-port surgery. Midline incision is widely used for mini-laparotomy [6], because it is easy to perform, results in minimal blood loss, provides a better surgical view, and is more convenient for closure than other types of incision [7]. However, a midline incision carries a high incidence of incisional hernia [8].

Incisional hernia is a commonly encountered complication after abdominal surgery, with a reported incidence of 10–32% [9, 10]. Incisional hernia diminishes a patient’s quality of life due to pain and discomfort [11]. Although minimally invasive surgery with a smaller incision and limited abdominal wall trauma is expected to reduce incisional hernia, the incidence of incisional hernia of this approach did not differ from that of open surgery [12, 13]. Several methods of abdominal closure have been reported to prevent incisional hernia after abdominal surgery. A running technique with long-lasting monofilament suture material is recommended to prevent incisional hernia [14]. However, monofilament suture has the disadvantage of being easily loosening and requiring a surgical knot, resulting in a potential risk of incisional hernia [15].

Barbed suture is a new type of suture material, first patented in 1964. It consists of standard monofilament suture with tiny barbs along its length that are designed to anchor the suture without the need for knots [16]. These new materials have become popular for skin and uterine closures during cesarean section, orthopedic surgery, and gastrointestinal surgery due to reduced tissue trauma and convenience [17–20]. In animal experiments, barbed sutures for fascia closure showed adequate tensile strength [21]. However, they are yet to be clinically adopted owing to insufficient evidence of their safety and efficacy as compared to those of conventional suture materials. Therefore, we propose a randomized controlled clinical trial (Barbed trial) to compare the outcomes of absorbable barbed sutures and conventional absorbable monofilament sutures for midline fascia closure in minimally invasive surgery for colorectal and gastric cancers.

### Objective {7}

This study aimed to evaluate the safety and efficacy of absorbable barbed sutures for midline fascia closure in minimally invasive surgery for colorectal and gastric cancers in comparison with conventional absorbable monofilament sutures.

### Study design {8}

**Fig. 1:**
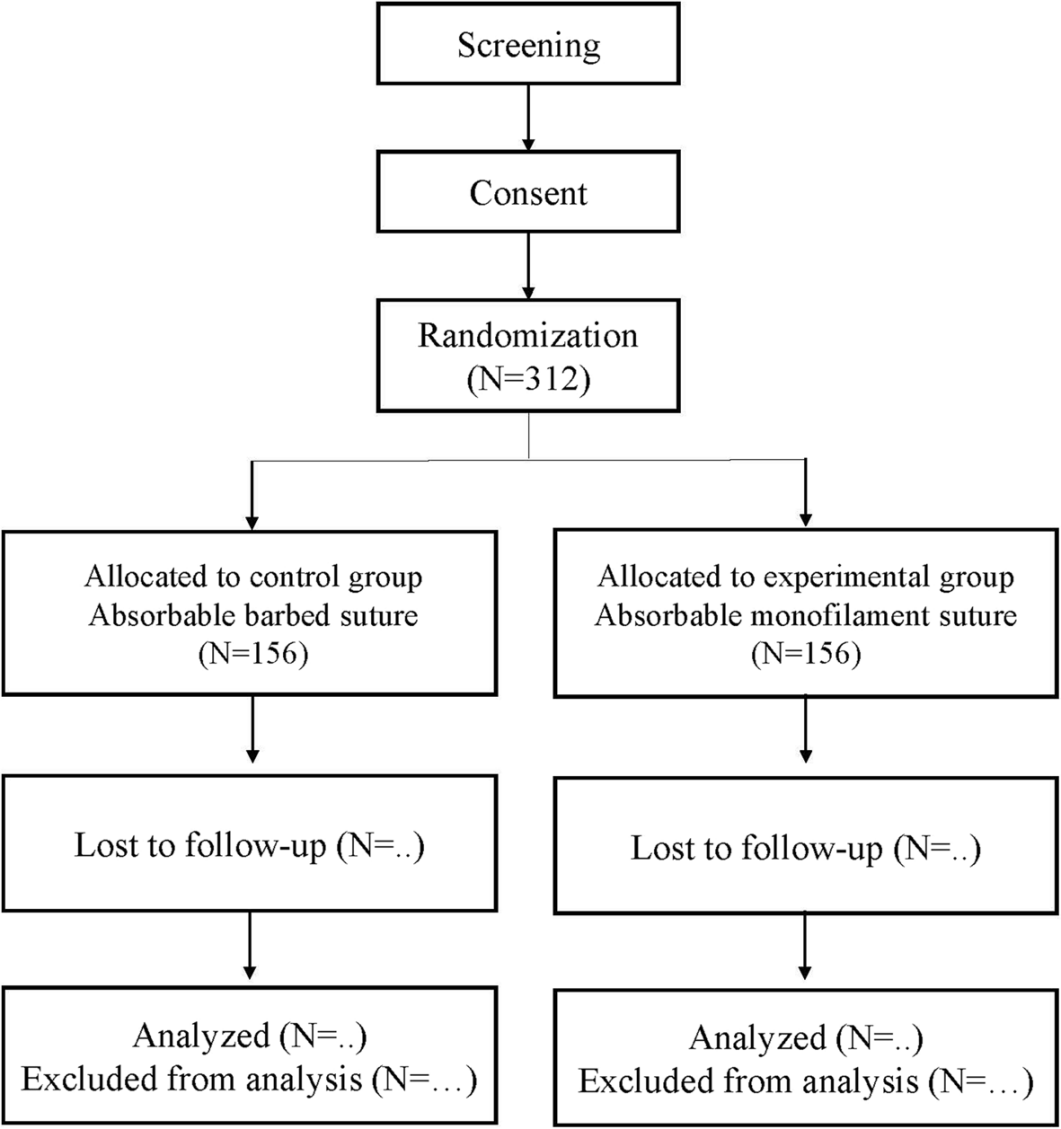
Control group : Absorbable monofilament suture

The Barbed trial is a prospective, superiority, single-center, randomized trial comparing the clinical outcomes of absorbable barbed sutures and monofilament sutures. A total of 312 patients will be enrolled and randomly allocated to use absorbable barbed or monofilament sutures for midline fascia closure in a 1:1 ratio. The surgical procedure used to close the midline fascia was standardized for both types of suture material. The investigator will examine the patients until discharge and at 6, 12, 18, 24, and 36 months postoperatively (Fig. 1). This study is ongoing at Jeonbuk National University Hospital and will last 3 years for each patient. This trial was registered with the Clinical Research Information Service of Korea.

## Method: participants, interventions, and outcomes

### Study setting {9}

This study will enroll participants who are scheduled to undergo minimally invasive gastric or colorectal cancer surgery at Jeonbuk National University Hospital. This study was approved and supported by the Institutional Review Board of Jeonbuk National University Hospital (CUH 2021-09-040).

### Eligibility criteria {10}

#### Inclusion criteria

The inclusion criteria were as follows: (1) patients who were diagnosed with colorectal or gastric adenocarcinoma, (2) patients who underwent laparoscopic and robotic radical surgery, and (3) patients who were older than 20 years of age.

#### Exclusion criteria

Patients would be excluded if they fulfill any of the following exclusion criteria:

- Patients who underwent open surgery
- Patients with possible distant metastasis in preoperative studies
- Patients who underwent past abdominal surgery
- Patients who had midline incision of less than 1 cm or longer than 10 cm
- Patients who had incision which is not located at the midline
- Patients who underwent emergency operation, palliative surgery, or ileostomy or colostomy formation
- Patients with cancer-related complications (perforation or abscess)
- Patients who participated in another clinical trial within the past 6 months

### Who will take informed consent? {26a}

The trial details will be explained in full to potential participants by investigators, and an informed consent form will be provided. Adequate time will be given to participants to consider their decision regarding trial participation. Subsequently, participants can sign the informed consent and they can withdraw at any time during the trial.

### Additional consent provisions for collection and use of participant data and biological specimens {26b}

Not applicable as no participant data and biological specimens were collected in this study.

### Interventions

#### Intervention description {11a}

Surgical procedures are performed under general anesthesia and by an experienced surgeon. To achieve standardization of suture technique, we provide surgeons with formal video of fascial closure using two suture materials. The location of the midline incision, which is supra-, trans-, or infra-umbilical mini-laparotomy, is determined according to the surgeon’s preference. At the end of surgery, fascia closure is achieved with absorbable barbed sutures (Stratafix®, Ethicon Inc., USA) for the study group and absorbable monofilament sutures (Maxon®, Covidien Inc., USA) for the control group. In all cases, the suture-to-wound length ratio is at least 4:1 for closure of the midline incision, and the inter-suture spacing is less than 1 cm. We avoid mass closure, which is performed with a suture bite, including all layers of the abdominal wall, except the skin. A single aponeurotic closure is applied to the midline incision in both groups. Subcutaneous tissue closure is not mandatory in this study. The skin can be approximated by using staples.

#### Criteria for discontinuing or modifying allocated interventions {11b}

There are no predetermined criteria for discontinuing or modifying the intervention assigned to participants. All individuals are participating on a voluntary basis, and they have the option to withdraw from the study at any time for any reason without facing any negative consequences.

#### Strategies to improve adherence to interventions {11c}

We will recruit patients who are scheduled to undergo surgery for gastric or colorectal cancer. By enrolling participants who require regular cancer screening, we anticipate that adherence to the study protocol will improve. Furthermore, participants will be informed of the significance of completing follow-up assessments.

#### Relevant concomitant care permitted or prohibited during the trial {11d}

All participants will receive standard postoperative management after surgery for gastric or colorectal cancer. For patients with wound infection, commercial dressing preparations or negative pressure wound therapy will be permitted.

#### Provisions for post-trial care {30}

There will be no provision for patients who participate in the trial. Post-trial care will follow the standards of care after gastric and colorectal cancer surgery.

### Outcomes {12, 18a}

#### Primary outcome

The primary outcome of this trial is the frequency of incisional hernia 3 years after surgery. We use the definition of incisional hernia according to the European Hernia Society: “any abdominal wall gap with or without bulge in the area of a postoperative scar perceptible or palpable by clinical examination or imaging” [22]. All patients will be examined using computed tomography to evaluate cancer recurrence during the study period. Therefore, the investigator will examine the occurrence of incisional hernia through physical examination and computed tomography scans at 6, 12, 18, 24, and 36 months after surgery in the outpatient clinic.

#### Secondary outcomes

Postoperative complications, including surgical site infection (SSI), pain, and quality of life, will be compared between the absorbable barbed suture and conventional absorbable suture groups.

**Fig. 2:**
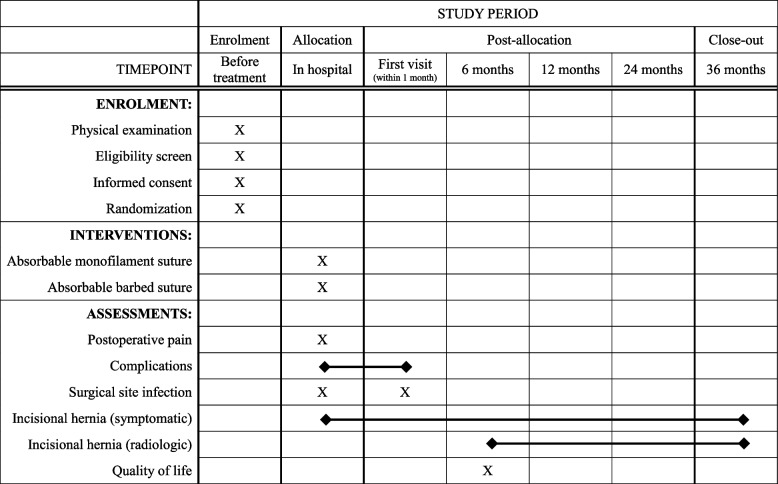
Experimental group : Absorbable barbed suture

Postoperative complications is defined as any adverse events that required additional pharmacological, interventional, or surgical management within 30 days after surgery. All postoperative complications were graded according to the Clavien-Dindo classification [23]. SSI is defined according to the criteria of the Centers for Disease Control and Prevention [24]. We divided SSI into superficial, deep incisional, and organ/space-related types occurring within 30 days after surgery. We will evaluate postoperative pain on postoperative days 1, 2, and 3 using a numeral rating scale. The verbal numeral rating scale ranged from 0 to 10 (0 = no pain and 10 = worst pain). To analyze the quality of life, the SF-36 and body image questionnaire will be used and documented by the patient 12 months postoperatively (Fig. 2). All outcomes will be evaluated by the investigators and they are specially trained for reliability.

### Participant timeline {13}

The participant timeline is shown in Fig. 2.

### Sample size {14}

We mainly have used absorbable monofilament sutures for fascia closure, and the overall incidence of incisional hernia was about 15%. Barbed suture began to be used for fascial closure in August 2020 during colorectal surgery. Before this randomized controlled trial, we conducted a pilot study of barbed suture for fascia closure. A total of 60 patients underwent fascia closure with barbed suture during laparoscopic colorectal surgery, and incisional hernia occurred in 2 patients (3.3%) within 1 year after surgery. Assuming additional patients with an incisional hernia during follow-up period, we determined possible incisional hernia rate to 5% in the barbed suture group for 3 years after surgery. Therefore, we hypothesized that the incidence of incisional hernia at 3 years after surgery will be lower in the barbed suture group than in the monofilament suture group. The sample size was calculated on the basis of this analysis plan, assuming that the incisional hernia rate of the barbed suture and monofilament groups at 3 years after surgery will be 5% and 15%, respectively. We calculated that 141 patients per group needed to achieve a two-sided α of 0.05 and statistical power of 80% using a chi-squared test. Assuming a 10% dropout rate, it is necessary to recruit 312 patients. Finally, 156 patients were assigned to the barbed suture and monofilament groups. The calculations were performed using G*Power 3.1.

### Recruitment {15}

Patients who are scheduled to undergo minimally invasive gastric or colorectal cancer surgery will be considered as candidates for trial enrolment in concordance with the inclusion and exclusion criteria. Informed consent will be obtained from all participants by investigators. We are confident that we can enroll a sufficient number of eligible patients, as our institution conducts over 150 surgeries for gastric cancer and 300 surgeries for colorectal cancer annually.

## Assignment of interventions: allocation

### Sequence generation {16a}

Eligible patients will be randomly allocated to use absorbable barbed or monofilament sutures for midline fascia closure in a 1:1 ratio. Stratified randomization was performed by medical statisticians who do not participate in this study. This randomization is based on an independent, computer-based sequence generated from the implementation of the dynamic algorithm with the cancer types as the stratifying variables.

### Concealment mechanism {16b}

To achieve randomization, a computer-generated sequence methodology will be used, which will ensure that both the randomization methodology and the allocation sequence are concealed from both the investigator and the participants.

### Implementation {16c}

An independent medical statistician will generate a stratified randomization list for allocation sequence. Investigators will enroll and assign participants to interventions.

## Assignment of interventions: blinding

### Who will be blinded {17a}

After the interventions are assigned, participants will be blinded. The results will be kept confidential from the participants throughout the entire trial. Surgeons will not be blinded because the intervention involves a surgical procedure.

### Procedure for unblinding if needed {17b}

As investigators are aware of the participant allocation, unblinding would not be required.

## Data collection and management

### Plans to promote participant retention and complete follow-up {18b}

Participants are patients with gastric or colorectal cancer who require regular follow-up to evaluate cancer recurrence. We expect that participants will be available for follow-up due to concerns of cancer recurrence.

### Data management {19}

The study data will be collected and recorded electronically, and stored securely on computers and hard drives. Only the investigators will have access to the participants’ files, and they will be the only ones authorized to make changes to the information. This will ensure that the data remains accurate and validated throughout the study.

### Confidentiality {27}

Datasets will be anonymized, and only summary data, which cannot identify individual participants, will be presented in the manuscript.

### Plans for collection, laboratory evaluation, and storage of biological specimens for genetic or molecular analysis in this trial/future use {33}

Not applicable as no biological specimens were collected in this study.

## Statistical methods

### Statistical methods for primary and secondary outcomes {20a}

A planned analysis will be performed after all patients have completed their 3-year follow-up. Categorical variables will be presented as numbers with percentages, and continuous variables will be presented as means with standard deviations or medians with interquartile ranges. The analysis will be performed using the intention-to-treat approach. The primary outcome will be analyzed using chi-squared and Kaplan-Meier tests. Risk factors for incisional hernia will be analyzed using the Cox regression analysis. Secondary outcomes will be tested using the chi-squared test for categorical variables and the *t*-test or Mann-Whitney test for continuous variables, as appropriate. The level of statistical significance will be set at *p* < 0.05.

### Interim analyses {21b}

We have no plans to conduct any interim analyses, since both interventions have low risk.

### Method for additional analyses (e.g., subgroup analyses) {20b}

Subgroup analyses will be performed on the type of cancer (gastric or colorectal cancer). Values of *p* < 0.05 will be considered statistically significant.

### Methods in the analysis to handle protocol nonadherence and any statistical methods to handle missing data {20c}

Loss to follow-up will be minimized due to the characteristics of cancer patients. If missing data is exceed 5% of any variable, multiple imputations will be used to handle missing values.

### Plans to give access to the full protocol, participant-level data, and statistical code {31c}

The full protocol and final datasets will be available from the corresponding author on reasonable request.

## Oversight and monitoring

### Composition of the coordinating center and trial steering committee {5d}

The authors will coordinate and steer this study.

### Composition of the data monitoring committee, its role and reporting structure {21a}

We do not have composition of the data monitoring committee.

### Adverse event reporting and harms {22}

Any adverse events that required additional pharmacological, interventional, or surgical management within 30 days after surgery will be collected and monitored. We regarded adverse events as postoperative complications and graded according to the Clavien-Dindo classification [23]. Adverse events will be treated according to the standards of care.

### Frequency and plans for auditing trial conduct {23}

The study data will be maintained in accordance with Good Clinical Practice requirements by the investigators. The original study data and information will be securely stored for a minimum of 5 years following the completion of the study. Data monitoring reports will be submitted to the ethics committee every 3 months.

### Plans for communicating important protocol amendments to relevant parties (e.g., trial participants, ethical committees) {25}

If the protocol needs to be modified, it will be reviewed again by the ethics committee, and, upon approval, the trial registry and protocol will be updated.

### Dissemination {31a}

The findings will be published in peer-reviewed journals and disseminated through scientific and academic conferences.

## Discussion

This is the first randomized controlled trial to compare absorbable barbed sutures with absorbable monofilament sutures for midline fascia closure in minimally invasive surgery for colorectal and gastric cancers.

The current guidelines recommend monofilament suture material for continuous closure of the midline incision [22]. However, absorbable monofilaments can stretch up to 30% of their length and slip easily, loosening the suture material [15]. At the end of the monofilament suture, it is necessary to create a surgical knot that can break and cause an incisional hernia. Barbed sutures were introduced several years ago. This novel suture material has a self-anchorage system that maintains tension and requires no surgical knots after the sutures are strained [25]. Although barbed sutures have been applied in uterine closure, arthroscopic surgery, and gastrointestinal surgery, they are not widely used for fascia closure due to the lack of evidence of its safety and efficacy.

In this trial, we aimed to determine which suture material is safer for patients who require mini-laparotomy in minimally invasive surgery. The strength of this study was its prospective and randomized design. If absorbable barbed sutures demonstrate comparable results to those of monofilament sutures, they might be suggested as an alternative suture material for abdominal fascia closure.

## Trial status

The study protocol was registered on December 23, 2021. The first patient was recruited on December 24, 2021. Recruitment is expected to end in December 2023.

## Data Availability

The datasets used or analyzed during the study are available from the corresponding author on reasonable request.

## Abbreviation

SSI: Surgical site infection
